# Assisted technology for daily living

**DOI:** 10.1186/2193-1801-3-S1-K2

**Published:** 2014-12-04

**Authors:** Kai-yu Raymond Tong

**Affiliations:** Division of Biomedical Engineering (BME), Department Electronic Engineering, The Chinese University of Hong Kong, Kragujevac, Hong Kong

Two assisted technologies for daily living have been developed recently by Prof. Raymond K.Y. Tong: Telecare system for ihome and Kinect for the elderly exercise. Telecare system is for monitoring the safety of single elderly at home, and Kinect system is designed for home-based exercise software for the elderly (named KineLabs).

## TeleCare

The home care service continues to grow as the overall population ages, and as the economic situation requires parents and other adults of the family all out for work and for longer hours. At the same time, Hong Kong, following the global trend, is shifting its care provision more and more from the expensive institutional-based model to the more economical community-based and family-based model. Accidents could happen when the elderly just alone at home and they might not be able to send out help-seeking alerts to their care-givers immediately, which could cause the risk of safety. An activity monitoring system would use various infrared sensors to monitor the activity patterns of the client in a non-obtrusive manner within an apartment. The system would prompt the user for response if certain patterns of vital signs and activities deemed critical appear. The proposed TeleCare technology for iHome would offer to the end-users as a mean to alert their care-providers when they are in certain critical situations. The deliverables would have impacts on the 500,000 elderly in Hong Kong who live singly or pseudo-singly (when other family members are all out for work), plus other vulnerable who are staying at home often by themselves, such as people with disabilities and chronic illness, summing to another 500,000 in the territory. Many of them would appreciate some forms of care service in case of emergency. This development facilitates care at home without requiring other family members and care-providers to stay home all the time.

A Telecare system has been developed using wireless passive infrared (PIR) motion detectors and a software program of Pattern of Daily Activities (PDA) which was run to collect daily pattern data and used these data to setup the threshold parameters in different areas. Each threshold parameter was also divided into four time zones (for example, mid-night: 00:00-05:59; morning: 06:00-11:59; afternoon: 12:00-17:59; evening: 18:00-23:59). These four time zones were defined to match different major activity pattern during a day. In the mid-night period, it covers the sleeping time; in the morning, it covers morning activities; in the afternoon, it may cover the time for taking a nap; in the evening, it covers the activities in the night.

## KineLabs

Integrating rehabilitation and fun together is a good experience for elderly and persons with motor disabilities, therefore we developed a new platform called "KineLabs". The Kinect is a new, exciting technology that provides integrated depth sensors to interact with 3D virtual environment. Moreover, the hardware price (including the Kinect sensor and PC/laptop) is affordable to many rehabilitation centres and home-users. Adopting the latest technology, this project aims at developing new training algorithms in order to benefit the elderly and persons with motor disability after stroke for rehabilitation and improvement of their quality of life. The project developed a software platform with three rehabilitation games that offers coordinated upper limb exercise, lower limb exercise and trunk balance training tasks such as making egg tart, cleaning the window panels of a tram, and even killing cockroaches. It also supports multi-players, and the platform is free for the public. During the training, 3D information of body segments could be captured and further analyzed by using our evaluation platform, such as range of motion and quality of motor control. Thus, our system can promote healthy ageing and interactive rehabilitation training at home, hospitals and elderly centres.

We have conducted a preliminary clinical trial, 19 stroke subjects were assessed by the evaluation platform. Stroke subjects had received a standard of 10 training sessions (1 hour per session). The clinical functional scores (Wolf motor function test and Fugl-Meyer Assessment (FMA)) showed functional improvement on their motor ability after 10-session of training.

